# Sudden Onset of Pseudotuberculosis in Humans, France, 2004–05

**DOI:** 10.3201/eid1407.071339

**Published:** 2008-07

**Authors:** Pascal Vincent, Alexandre Leclercq, Liliane Martin, Yersinia Surveillance Network, Jean-Marie Duez, Michel Simonet, Elisabeth Carniel

**Affiliations:** * Institut National de la Santé et de la Recherche Médicale (INSERM), Lille, France; † Université Lille 2, Lille; ‡ Institut Pasteur de Lille, Lille; § Institut Pasteur, Paris, France; ¶ Centre Hospitalier du Bocage, Dijon, France; 1 These authors contributed equally to this article.; 2 These authors contributed equally to the supervision of this work.

**Keywords:** Yersinia pseudotuberculosis, disease outbreaks, France, dispatch

## Abstract

Cases of *Yersinia pseudotuberculosis* infection increased in France during the winter of 2004–05 in the absence of epidemiologic links between patients or strains. This increase represents transient amplification of a pathogen endemic to the area and may be related to increased prevalence of the pathogen in rodent reservoirs.

*Yersinia pseudotuberculosis* is an enterobacterial pathogen able to grow at low temperatures. It is widespread in the environment (e.g., water, plants), which is the source of contamination for mammals (especially rodents and their predators) and birds (*1*,*2*). Although most cases of human infection are sporadic, outbreaks have occurred in Japan (*3*,*4*), Russia (*5*,*6*), and Finland (*7*,*8*), mainly associated with unchlorinated drinking water or contaminated vegetables. In France, similar increases in case numbers had not been noted until the winter of 2004–05.

## The Study

In early January 2005, the French *Yersinia* National Reference Laboratory (YNRL) received 3 *Y. pseudotuberculosis* strains isolated by the same laboratory in Dijon over a 1-week period: 2 from fecal samples of 2 children attending the same daycare center and 1 from the blood of a 65-year-old woman. During the same month, 8 additional strains were isolated from persons in other parts of the country by the *Yersinia* Surveillance Network (based on voluntary participation of 88 hospital-based and private-sector medical laboratories throughout France).

In view of this unusually high number of *Y. pseudotuberculosis* isolations over a short period, the YNRL alerted France’s national disease surveillance network, the Institut de Veille Sanitaire, which thereafter performed an epidemiologic investigation. In early February, a request was mailed to all member laboratories in the *Yersinia* Surveillance Network and all 95 microbiology laboratories in university medical centers, asking them to report any recent cases of *Y. pseudotuberculosis* infection. A reminder letter was mailed to all laboratories that had not replied within 1 month of the initial communication. Moreover, from February through April 2005, a total of 76 general medical center laboratories were contacted by telephone and asked to provide the YNRL with any relevant information and/or isolates. Overall, 27 cases of culture-confirmed *Y. pseudotuberculosis* infections were spontaneously reported or actively retrieved (Table 1).

**Table 1:** Relevant characteristics of 27 patients with *Yersinia pseudotuberculosis* infection, France, winter 2004–05*

Age, y	Sex	Risk factors	Main clinical signs/symptoms	Site of organism isolation	O serotype	Illness outcome
0.8	M	None	Diarrhea	Feces	I	Recovery
1	M	None	Diarrhea	Feces	III	Recovery
2	F	None	Diarrhea	Feces	I	Recovery
6	M	None	Diarrhea	Feces	I	Recovery
9	F	None	Diarrhea	Feces	I	Recovery
17	M	Multiple injuries (motorcycle accident)	Postsurgical infection†	Blood	I	Recovery
36	F	HIV infection	Diarrhea, mesenteric adenitis	Feces	I	Recovery
44	F	Bone marrow transplantation	Fever	Blood	I	Death
51	F	Sickle cell anemia, cirrhosis	Diarrhea, esophageal variceal bleeding	Blood	I	Death
59	M	Cirrhosis	Fever, esophageal variceal bleeding	Blood	I	Recovery
61	M	Therapeutic aplasia (colorectal cancer)	Fever, abdominal pain	Blood	I	Death
64	M	Abdominal aortic aneurysm	Abdominal pain	Artery biopsy	I	Recovery
65	F	Myeloma	Fever, septic shock	Blood	I	Death
70	M	Cirrhosis	Fever	Blood	I	Recovery
71	M	Unknown	Abdominal pain	Blood	NA strain	Recovery
71	M	Diabetes, steroid receipt (for retroperitoneal fibrosis)	Fever, diabetes decompensation	Blood	I	Recovery
72	M	Kidney transplantation	Fever	Blood	I	Recovery
74	M	Diabetes	Fever, abdominal pain	Blood	NS	Recovery
75	M	Viral hepatitis C infection	Fever	Blood	I	Recovery
76	M	Cirrhosis	Fever	Blood	NS	Recovery
78	M	Calcific aortic stenosis	Fever, acute heart failure	Blood	I	Recovery
78	M	Diabetes	Fever, septic shock	Blood and artery biopsy	I	Death
79	M	Metastatic colorectal cancer	Fever, respiratory distress syndrome	Blood	I	Death
80	M	Cerebrovascular accident	Fever, cardiogenic shock	Blood	NA strain	Recovery
81	F	Cirrhosis	Fever	Blood	I	Recovery
82	M	Diabetes	Fever, septic shock	Blood	NA strain	Death
83	M	Diabetes	Fever	Blood	I	Recovery

*NA, nonagglutinable; NS, not sent to *Yersinia* National Reference Laboratory.  †This patient’s signs/symptoms began after hospitalization; all other patients’ signs/symptoms began before hospitalization.

**Figure 1:**
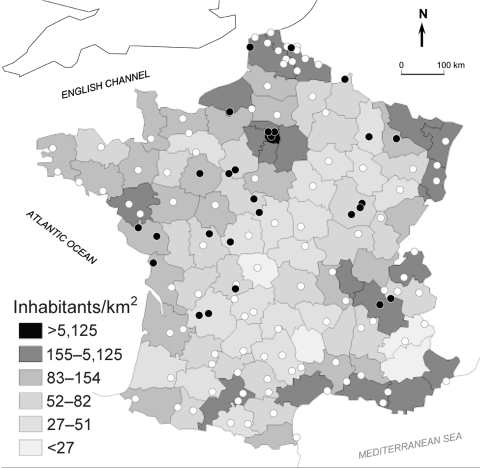
Map of France, showing spatial distribution of *Yersinia pseudotuberculosis* infections during the winter of 2004–05. Black circles, patients' residences; open circles, cities with medical laboratories that stated that they had not isolated any *Y. pseudotuberculosis* from clinical specimens.

Case reports of *Y. pseudotuberculosis* infection peaked in January 2005. A food-exposure analysis for the first 10 patients did not identify a potential common food source, so a food survey was not performed for subsequent cases. The pseudotuberculosis cases occurred in 19 different counties throughout France, not necessarily the most populated ones (Figure 1). Data on lifestyle and living conditions were obtained for all but 7 patients, of whom 3 had died and 4 (including 2 children) were lost to follow-up. Of the 22 adults, 13 lived in small towns (<5,000 inhabitants) and 5 lived in rural villages (<500 inhabitants). All but 4 lived in houses, as opposed to apartments. Of the 17 adults whose lifestyle was investigated in detail, 14 had a dog, hunted, gardened, and/or grew their own vegetables. In contrast, all 5 children lived in urban areas (>50,000 inhabitants), compared with only 3 of the 22 adults; 4 of the children lived in apartments.

Of the 27 strains isolated, 25 were sent to the YNRL for characterization. All but 4 strains belonged to serotype I, the most common serotype in France. Pulsed-field gel electrophoresis after *Spe*I digestion of genomic DNA showed that (with the exception of the isolates from the 2 children attending the same daycare center) the DNA fingerprints of the 16 other isolates sent to the YNRL during the peak period were all distinct, even when the strains were isolated from the same county (data not shown).

## Conclusions

This episode of increased case numbers differs from episodes reported in the literature by the nationwide distribution of cases, the absence of a locally defined cluster, the genetic diversity of the isolates, the predominance of rural residence for patients, and the dominant clinical presentation of septicemia (*3*–*8*). Because the cases were not related to the consumption of a food product sold nationally (e.g., by a supermarket chain), the unknown origin of this phenomenon raises the question of an emerging risk in a new epidemiologic situation.

Of the 27 patients, 19 lived in a strip of land that stretches from northern France to the Atlantic coast and corresponds to the flyways of small migratory birds. Hence, the avian introduction of strains into the country would have been a possible scenario, as has already been suspected for an epizootic of pseudotuberculosis in an American wildlife park (*9*). Indeed, during the winter of 2004–05, France witnessed a large and unexpected invasion of Bohemian waxwings (*Bombycilla garrulus*) (*10*), a species known to migrate from circumpolar areas where pseudotuberculosis is endemic. At that same time, 3 pseudotuberculosis outbreaks were reported in Siberia (*11*). However, the genetic diversity of the strains isolated from the patients in France and the absence of PCR amplification of the superantigen-encoding gene *ypm* gene (*12*) (which is highly prevalent in Far Eastern strains [*1*]) do not support bird-borne arrival in France of a *Y. pseudotuberculosis* clone from Russia or the Far East.

**Figure 2:**
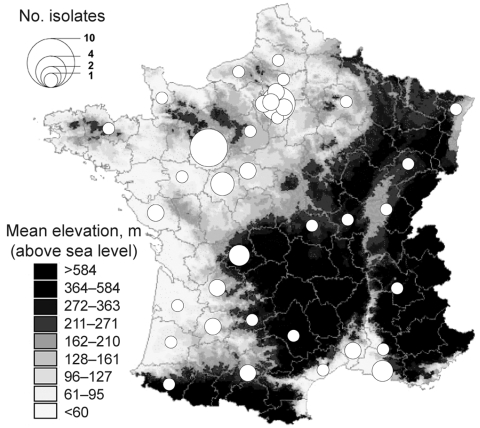
County distribution, France, of *Yersinia pseudotuberculosis* isolated from human blood and reported to the *Yersinia* National Reference Laboratory over the 16 years preceding the winter of 2004–05. The number of isolates is represented by proportionally sized circles arbitrarily located at the center of the counties.

These cases occurred in areas where other human cases of *Y. pseudotuberculosis* septicemia had been diagnosed (albeit at a much lower rate) in the past 16 years (Figure 2). Exacerbation of a preexisting epidemiologic situation is quite likely. Most previous cases of *Y. pseudotuberculosis* septicemia also concerned inhabitants of low-altitude plains (Figure 2), mainly in rural areas with extensive agriculture zones, which provide favorable habitats for small mammals. Cases were frequently (27.1%) reported in 5 central-western counties of France, where just 4.6% of the population live and incidence of *Francisella tularensis* infection (tularemia) is high (27.3%) (www.invs.sante.fr/surveillance/tularemie/donnees.htm). Moreover, 40 cases of tularemia (with incidence peaks in summer and autumn) were reported to the national surveillance system in 2004 (notification of the disease has been obligatory since 2002), whereas only 8 to 19 cases per year had been reported over the preceding and following periods. Like *Y. pseudotuberculosis*, *F. tularensis* is known to have a rodent reservoir. Hence, the spatial and temporal correlations between human tularemia and pseudotuberculosis in France over recent years suggest the sudden expansion of a common reservoir in 2004.

Our analysis of the temporal distribution of human *Y. pseudotuberculosis* septicemia cases over the past 16 years showed a peak every 5 years (Table 2). This finding is reminiscent of human hantavirus infections, which have been linked to cyclical oscillations in the vole population (the virus reservoir). Taken as a whole, these data suggest that a rodent reservoir, mainly in rural areas, could have suddenly increased in the spring of 2004, thus increasing the risk for human transmission of *Y. pseudotuberculosis* and *F. tularensis* over the following months.

**Table 2:** Temporal distribution of receipt of*Yersinia pseudotuberculosis* blood isolates, France

Period	Monthly isolates*												Annual isolates
	Sep	Oct	Nov	Dec	Jan	Feb	Mar	Apr	May	Jun	Jul	Aug	
1988–89	0	0	0	0	1	1	2	1	0	0	1	0	6
1989–90	0	0	1	0	1	2	1	0	1	1	0	0	7
1990–91	0	1	0	0	1	1	0	4	0	0	0	0	7
1991–92	0	0	0	1	0	1	1	0	1	0	0	0	4
1992–93	0	0	0	0	1	1	0	1	0	0	0	0	3
1993–94	0	0	0	0	0	0	3	0	2	0	0	0	5
1994–95	0	0	0	0	1	3	1	2	0	0	0	1	8
1995–96	0	0	0	0	1	0	3	0	0	2	0	1	7
1996–97	0	0	0	0	0	1	0	0	0	0	0	0	1
1997–98	0	0	0	0	2	0	0	0	0	0	0	1	3
1998–99	0	0	0	0	0	0	0	1	0	1	0	0	2
1999–00	0	0	0	1	0	2	1	0	0	2	1	1	8
2000–01	0	0	0	0	1	0	0	1	0	2	0	0	4
2001–02	0	0	1	1	0	0	0	0	1	0	0	0	3
2002–03	0	0	0	0	0	1	1	0	0	0	0	0	2
2003–04	0	0	0	0	0	1	4	1	0	2	0	0	8
2004–05†	0	0	0	0	4	4	5	5	0	0	0	0	18
2005–06	0	0	0	0	0	2	2	0	0	0	0	0	4
1988–2006‡	0	1 (0.06)§	2 (0.12)	3 (0.18)	9 (0.53)	16 (0.94)	19 (1.12)	11 (0.65)	5 (0.29)	10 (0.59)	2 (0.12)	4 (0.24)	82 (4.82)

*Received by the French *Yersinia* National Reference Laboratory since September 1988. To encompass the whole cold season, isolates are presented in 12-month periods from September to August. †Period of increased number of cases. ‡Excludes 2004–05. §Numbers in parentheses are mean monthly value over the 17 years in which case numbers were not increased.

Rodent populations tend to increase with ongoing changes in agricultural practices, e.g., removal of farmland hedges (which provide shelter for the rodents’ predators) and reduction in pesticide use. Hence, the dynamics of the wild rodent population and reduction in pesticide use may represent a useful predictive marker for the occurrence of new outbreaks. The surveillance and control of the small mammal population might help limit the incidence of pseudotuberculosis and other wild rodent–borne diseases of humans in France.
